# Coverage of iron and folic acid supplementation in India: progress under the Anemia Mukt Bharat strategy 2017–20

**DOI:** 10.1093/heapol/czac015

**Published:** 2022-02-28

**Authors:** William Joe, Rinju, Narendra Patel, Ruby Alambusha, Bharati Kulkarni, Kapil Yadav, Vani Sethi

**Affiliations:** Population Research Centre, Institute of Economic Growth, Delhi University Enclave (North Campus), Delhi 110007, India; Institute of Economic Growth, Delhi University Enclave (North Campus), Delhi 110007, India; Institute of Economic Growth, Delhi University Enclave (North Campus), Delhi 110007, India; Institute of Economic Growth, Delhi University Enclave (North Campus), Delhi 110007, India; Clinical Division, National Institute of Nutrition, Jamai Osmania PO, Hyderabad 500007, India; Centre for Community Medicine (CCM), All India Institute of Medical Sciences (AIIMS), New Delhi 110029, India; Nutrition, UNICEF India, 73 Lodi Estate, New Delhi 110003, India

**Keywords:** Anemia Mukt Bharat, aneamia, iron and folic acid, AMB index, pregnant women, adolescents

## Abstract

High prevalence of anaemia is a severe public health problem in India. In 2018, India launched the Anemia Mukt Bharat (AMB) strategy that focuses on six beneficiary groups for coverage, six institutional mechanisms for health system strengthening and six programmatic interventions to accelerate reductions in anaemia prevalence. This paper uses the Health Management Information System data (2017–18 to 2019–20) to examine gains in IFA coverage across Indian states. A coverage-based AMB index is computed to review performance across states. After the launch of AMB strategy, the Iron and Folic Acid (IFA) supplementation coverage between 2017–18 and 2019–20 has increased for all beneficiary groups [pregnant women from 78% to 90%; lactating mothers from 34% to 49%; school going adolescent girls (boys) from 23% to 40% (21% to 42%); out-of-school adolescent girls from 6% to 23%; children 5–9 years from 8% to 3% and children 6–59 months from 7% to 15%]. Coverage was relatively low for target groups being served through a multi-departmental convergence mechanism (health and other departments such as education department for schools or women and child development department for *Anganwadi* centres) than compared to those served by health department alone. However, no major gender disparities are noted in the coverage of IFA supplementation among school-going girls and boys. Bulk of the variations in coverage is attributable to state-specific differences. Training and sensitization workshops for state and district officials are found to be associated with increased coverage across beneficiary groups. The paper argues that despite following international best practices in the field, it is important to harness synergy in programme implementation across line departments to eliminate coverage inefficiencies.

**Key messages**

- The IFA supplementation coverage has increased across all beneficiary groups after the implementation of Anemia Mukt Bharat (translated as Anemia Free India) strategy.
- There is no evidence of gender disparities in the coverage of IFA supplementation among school-going girls and boys.
- Association in coverage improvements across beneficiary groups is more robust if the source of IFA supplementation (such as health facilities/AWC/schools) is same.
- AMB trainings and sensitization workshops has a favourable impact on increasing IFA supplementation coverage across states.

## Introduction

Anaemia is a severe public health problem widely prevalent across several low- and middle-income countries. The global burden of disease studies show that in 2010 one-third (32.9%) of the global population was anaemic and this condition accounted for 8.8% share in disabilities from all health conditions (Kassebaum *et al.*, 2014). The estimates also reveal that more than one-third (37.5%) of the global burden is borne by South Asia. Iron deficiency is a major reason of anaemia (Kassebaum *et al.*, 2014; Chaparro and Suchdev 2019; Sarna *et al.*, 2020), and accordingly, iron supplementation is widely regarded as an important strategy to reduce prevalence of anaemia among pregnant women (PW) and children (Sachdev and Gera 2013; Pasricha *et al.*, 2013). However, policies and programmes on iron supplementation coverage across developed countries have varied experiences. In particular, the coverage levels are influenced by programme design, approach towards community-based delivery, training of frontline workers, counselling of beneficiaries and a continuous supply of IFA supplements (Siekmans *et al.*, 2018). Given the relevance of such policies, this paper engages with the case of India and examines whether the changes in IFA supplementation coverage are associated with the revamped strategy referred to as the Anemia Mukt Bharat (AMB) or Anemia Free India campaign.

India contributes to bulk of the South Asian burden of anaemia prevalence because of both a large population size and high prevalence across age–sex groups. The National Family Health Survey (NFHS 2015–16) of India estimates that the prevalence of anaemia is 58% among children (6–59 months), 29% among adolescent boys, 54% among adolescent girls (15–19 years), 53% among women of reproductive age (WRA), 50% among PW and 58% among lactating mothers (IIPS and ICF 2017). The situation has worsened during NFHS 2019–21 with particularly higher increase in prevalence among children (6–59 months) and PW (MoHFW, 2021). Although, India has implemented IFA tablets supplementation programmes for pregnant women since the 1970s but these programmes were affected by multiple challenges and bottlenecks in supplies and coverage (Vijayaraghavan *et al.*, 1990; Kapil *et al.*, 2019a). Consequently, India did not experience any discernible change in anaemia prevalence across various age–sex groups. Recognizing these intricacies, the anaemia control programme was revamped in 2018 with past learnings and international best practices and re-launched as AMB (Anemia Free India) strategy to overcome fundamental challenges including programme financing, implementation and last mile delivery.

AMB aims to achieve the POSHAN *Abhiyaan* (Prime Minister’s Overarching Scheme for Holistic Nutrition Mission) targets of reducing prevalence of maternal and child anaemia in India by 3 percentage points per year (Paul *et al.*, 2018). Consistent with international best practices, AMB is essentially a refinement of strategies that also builds on the learning and experience from previous programmes including the National Iron Plus Initiative (NIPI). The AMB is thus essentially conceptualized as an intensified version of NIPI and puts forth a 6 × 6 × 6 strategic approach emphasizing on six beneficiary groups, six institutional mechanisms and six interventions (MoHFW, 2018; Paul *et al.*, 2018). The six beneficiaries of AMB are (1) children (6–59 months), (2) children (5–9 years), (3) adolescent boys and girls (10–19 years), (4) WRA (15–49 years), (5) PW and (6) lactating mothers. To bolster programme implementation and accountability, six institutional mechanisms are envisaged viz. (1) intra-ministerial coordination, (2) national AMB unit, (3) convergence with other ministries, (4) strengthening of supply chain and logistics and (5) development of AMB dashboard (www.anemiamuktbharat.info) and digital portal are envisaged. Finally, the six key interventions under AMB are as follows: (1) prophylactic IFA supplementation, (2) deworming, (3) intensified year-round behaviour change communication campaigns including ensuring delayed cord clamping in newborns, (4) testing of anaemia using digital methods and point of care treatment, (5) mandatory provision of IFA-fortified foods in public health programmes and (6) redressal of non-nutritional causes of aneamia in endemic pockets with special focus on malaria, haemoglobinopathies and fluorosis.

Previous assessments of anaemia control programmes noted that a dysfunctional supply chain and poor last mile delivery were among the important shortcomings of the IFA supplementation efforts (Vijayaraghavan *et al.*, 1990; Kapil *et al.*, 2019a). The AMB, therefore, specifically focuses on these issues and adopts a holistic approach to address systemic concerns through improved budgeting and planning. The success of such strategies can be gauged through key programmatic achievements such as improvements in coverage related key performance indicators. This paper aims to examine the status of IFA supplementation coverage in India for the selected beneficiary groups to provide insights on coverage improvements after the launch of the AMB strategy.

Using publicly available data from the Health Management Information System (HMIS) and AMB dashboard (www.anemiamuktbharat.info), the paper examines the coverage across Indian states and union territories (UTs) and ranks their performance during FY 2017–18 and 2019–20. The analysis has three specific objectives as follows: (1) to assess IFA coverage across beneficiary groups, (2) to examine IFA coverage across states and districts and (3) to study gender difference in IFA coverage among children and adolescents. Finally, we also utilize the information on AMB-related training and capacity building workshops conducted across states and use this as a treatment to understand whether a proactive programme implementation can also be associated with better coverage.

It may be noted that, similar to maternal and child health services, the AMB services were severely disrupted because of coronavirus disease 2019 (COVID-19) pandemic (Singh *et al.*, 2021). The general disruption of health care as well as school education services was initially because of the COVID-19-related lockdown protocols but full restoration of services, particularly in schools, has not resumed even long after the lockdown period (Ghate *et al.*, 2020; Debbarma and Durai 2021). The frontline healthcare workers were also engaged in COVID-19 services across states and districts (Nanda *et al.*, 2020). Therefore, while examining coverage improvements under AMB, it is important that the assessment avoids contamination of coverage-related insights because of COVID-19 disruptions. This paper, accordingly, restricts the scope of analysis to the pre-pandemic years of 2017–18 to 2019–20 for analysing the trends and patterns in coverage of IFA supplementation among children, adolescents, and pregnant and lactating mothers in India after the launch of AMB.

## Data and methods

The study is based on publicly available data downloadable from the AMB dashboard (www.anemiamuktbharat.info) and accessed on 29 November 2020. The dashboard is a one-stop shop for programme and coverage statistics. The HMIS portal (https://nrhm-mis.nic.in/SitePages/Home.aspx and https://hmis.nhp.gov.in/#!/) provides information on the number of different target beneficiaries. Under AMB IFA supplementation, pregnant and lactating mothers are provided with IFA red tablets whereas adolescent girls and boys in schools are provided with IFA blue tablets. Children aged 6–59 months are provided IFA syrup while those aged 5–9 years are provided IFA pink tablets. The dosage and composition of the IFA supplements are available in the AMB operational guidelines. The indicators specific to each group of beneficiaries are as follows: HMIS 1.2.4—percentage of eligible PW who received at least 180 IFA tablets in reporting month at the antenatal contact point; HMIS 9.9—percentage of children (6–59 months) who received at least 8 doses of IFA syrup in reporting month; HMIS 22.1.1—percentage of school-going adolescents (10–19 years) girls and boys, eligible under Weekly Iron and Folic Acid Supplementation (WIFS) programme, who received at least four blue coloured IFA tablets in reporting month; HMIS 23.1 + 23.3—percentage of children (5–9 years) in schools + out of school under WIFS junior who received at least four pink coloured IFA tablets in reporting month.

We compute the AMB index that is defined as the simple average of the coverage indicators across beneficiary groups. The numerators of the index were from HMIS standard reports and the denominators were extracted from AMB dashboard. The numerators and denominators account for monthly consumption frequency for indicators related to children: 6–59 months, 5–9 years, adolescents 10–19 years, PW and lactating mothers. It may be noted that a coverage statistic of 95% is considered as the ceiling as any estimate beyond this is highly improbable and may indicate reporting errors. The states and UTs are individually ranked on the basis of the AMB index, whereby the state/UT with highest index value is ranked first.

The spatial distribution of the average IFA supplement coverage change is also analysed. Descriptive statistics are reported as numbers and percentage for categorical data and mean ± standard deviation (SD) for state- and district-level coverage of IFA supplement. A correlation matrix was constructed to understand the association in coverage of IFA supplement across all beneficiary groups after the implementation of the programme. Spatial clustering patterns are presented for gender difference in coverage between adolescent boys and girls and difference between out-of-school adolescent girls and school-going adolescent girls. The NFHS data for the years 2015–16 and 2019–21 are also used to compare the levels and changes in IFA supplementation coverage among PW and children (IIPS and ICF 2017; MoHFW, 2021).

Utilizing the hierarchical nature of the data, multilevel linear regression models are used to analyse state- and district-level random effects in IFA supplementation. Two models were constructed for the analysis. The first model adjusts for state-level variables, and the second model further adjusts for district-level variables, simultaneously. The variance partition coefficient (VPC) was calculated for the state and district levels (Rabe-Hesketh and Skrondal 2008). In the simplest two-level case, the variation can be partitioned into a higher level component based on differences between higher-level units and a lower-level residual component. These higher level units are the sampling units. Such a model with only one random variable at each level is known as a variance components model (Browne *et al.*, 2005; Leckie and Charlton 2013).

Finally, difference-in differences (DD) method is applied to compare the changes in coverage of IFA supplementation over time between states that had conducted training and sensitization workshops for the AMB strategy (the treatment group) and states that had not (the comparison group). The DD approach allows correcting for any differences between the treatment and comparison groups that are constant over time (Khandker *et al.*, 2009; Villa, 2016). In other words, given a two-period setting where *t* = 0 before the AMB strategy (2017–18) and *t* = 1 after strategy implementation (2019–20), and letting *Y_t_^T^* and *Y_t_^C^* be the respective outcomes (IFA supplementation coverage) for a given beneficiary group from treatment and comparison groups in time *t*, the DD method estimates the average impact of training and sensitization workshops as follows:

(1) $${\rm{DD}} = {\rm{E}}\left( {{Y}_{\textit{1}}^{T} - {Y}_0^{T}{|T} = 1} \right) - {\rm{ E}}\left( {{Y}_{\textit{1}}^{C} - {Y}_0^{C}{|}{{T}_{\textit{1}}} = 1} \right)$$

In Equation (1), *T_1_* = 1 denotes treatment or the presence of the training and workshops at *t* = 1, whereas *T_1_* = 0 denotes comparison areas.

All analyses were performed using Stata 15.0 and ArcGIS software.

## Results

Table 1 reports the status of IFA supplement coverage across beneficiary groups for the years 2017–18, 2018–19 and 2019–20. Following the launch of the AMB strategy, coverage improvements are prominent across all the beneficiary groups. IFA supplementation coverage is highest among PW (90.3% in 2019–20) and lowest among children aged 6–59 months (14.9% in 2019–20). Coverage improvements (in percent) among children aged 5–9 years was 3.8 times in 2019–20 compared to the 2017–18 levels. Similarly, coverage improvements among out-of-school adolescent girls were 3.5 times in 2019–20 compare to the 2017–18 level. Prior to the launch of the AMB strategy, the coverage rate was marginally higher among adolescent girls (22.6%) in comparison to adolescent boys (21.1%). In 2019–20, however, boys have marginally higher coverage of IFA supplementation. The coverage level is higher among school-going adolescent girls (39.7%) than out-of-school adolescent girls (22.7%). An important aspect to note is the increased quantum of beneficiaries in absolute terms. The IFA supplementation now covers 26 million PW, 13 million lactating mothers, 17 million under 5 children, and 73 million school-age children and adolescents (5–19 years).

**Table 1.** Coverage of IFA supplementation under AMB, India 2017–18 to 2019–20

Beneficiary groups	2017–18	2018–19	2019–20
Children (6–59 months)	6.6%	8.3%	14.9%
	8.0 million	9.8 million	17.2 million
Children (5–9 years)	8.0%	14.9%	30.2%
	8.4 million	14.1 million	27.6 million
School-going adolescent girls	22.6%	29.1%	39.7%
	11.2 million	14.8 million	19.4 million
School-going adolescent boys	21.1%	26.6%	41.6%
	10.8 million	13.4 million	20.2 million
Out-of-school adolescent girls	6.4%	12.1%	22.7%
	2.3 million	3.2 million	5.3 million
Pregnant women	77.7%	84.8%	90.3%
	22.3 million	24.4 million	26.2 million
Lactating mother	34.4%	41.6%	49.0%
	9.0 million	10.7 million	13.0 million

Source: Authors based on HMIS and AMB dashboard (www.anemiamuktbharat.info).

**Figure 1.**
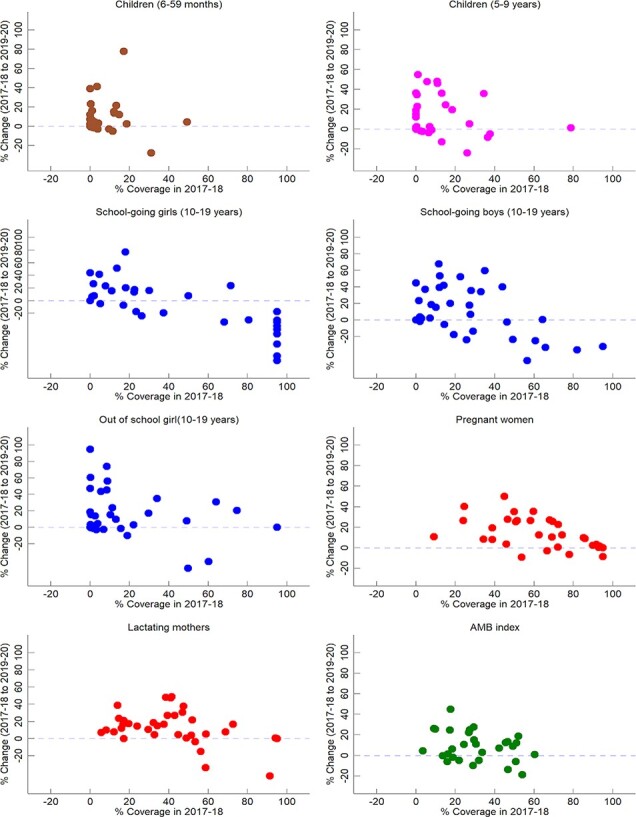
Association between change in IFA supplementation coverage and base-level coverage across states, India 2017–18 to 2019–20

*Source*: Authors based on HMIS and AMB dashboard (www.anemiamuktbharat.info).

Coverage improvements are usually higher when the base-level coverage is lower. Figure 1 shows the patterns in change in coverage (between 2017–18 and 2019–20) across states vis-à-vis base-level coverage in 2017–18. It is discernible that changes in coverage levels are relatively higher among states that had initially lower base level coverage in 2017–18. Since most of the states continue to have lower base-level coverage, therefore, major increments in coverage can be expected in IFA supplementation. The increments can be particularly higher among children and lactating mothers.

Table 2 presents the AMB index defined as a simple average of IFA supplementation coverage across the seven beneficiary groups. In 2017–18, Andhra Pradesh (60.4%), Sikkim (54%), Pondicherry (52.1%), Gujarat (51%) and Tamil Nadu (50.8%) reported the highest AMB index values for IFA supplementation. On the other hand, Nagaland (3.5%), Uttarakhand (9.2%), Bihar (9.7%), Arunachal Pradesh (10.7%) and Tripura (15.9%) reported the lowest index values. Whereas, in 2019–20, Pondicherry (70.9%), Gujarat (63.1%), Himachal Pradesh (62.4%), Andhra Pradesh (61.3%) and Madhya Pradesh (60.0%) reported the highest index values and Nagaland (7.9%), Tripura (9.9%), Manipur (13.2%), Meghalaya (17.1%) and Jammu and Kashmir (17.7) reported the lowest values.

**Table 2.** AMB IFA supplementation coverage index values for seven beneficiary groups, 2017–18 to 2019–20

States	AMB index 2017–18	AMB Index 2019–20	Change (2017–18 to 2019–20)
Andaman & Nicobar Islands	NA	86.6	NA
Puducherry	52.1	70.9	18.8
Gujarat	51	63.1	12
Himachal Pradesh	17.7	62.4	44.7
Andhra Pradesh	60.4	61.3	0.9
Madhya Pradesh	46.7	60	13.3
Goa	NA	59.4	NA
Maharashtra	49.3	58.2	8.9
Assam	45.6	58	12.4
Haryana	29.4	56.8	27.4
Dadra & Nagar Haveli	27.3	52.2	24.9
Karnataka	42.2	49.3	7.1
West Bengal	27	49.1	22.1
Chhattisgarh	NA	46.2	NA
Tamil Nadu	50.8	44.7	−6.1
Orissa	29.6	44.5	15
Uttar Pradesh	17.2	41.7	24.5
Telangana	30.5	41.4	11
Daman & Diu	33.6	36.3	2.7
Uttarakhand	9.2	35.3	26.1
Bihar	9.7	35.2	25.5
Jharkhand	24.3	35.1	10.8
Sikkim	54	35.1	−19
Punjab	46.9	33.1	−13.8
Chandigarh	NA	31.5	NA
Mizoram	32	27.1	−4.9
Rajasthan	18.5	24.6	6.1
NCT of Delhi	NA	21.7	NA
Kerala	29.1	19.1	−10
Jammu & Kashmir	16.3	17.7	1.4
Lakshadweep	21.9	17.1	−4.8
Meghalaya	18.8	17.1	−1.8
Manipur	13.6	13.2	−0.4
Tripura	15.9	9.9	−6
Nagaland	3.5	7.9	4.4
Arunachal Pradesh	10.7	NA	NA
India	25.2	41.2	15.9

Source: Authors based on HMIS and AMB dashboard (www.anemiamuktbharat.info).

Table 2 also compares the change in AMB index value between 2017–18 and 2019–20 across states/UTs. In 27 states/UTs, there is an increase in the AMB index value between these two years. The highest increase in mean coverage across beneficiary groups is experienced by Himachal Pradesh (44.7%) followed by Haryana (27.4%) and Bihar (25.5%). Altogether, 9 out of 36 states/UTs have experienced a decline in mean coverage as revealed by the AMB index values. These states/ UTs are as follows: Sikkim (−19%), Punjab (−13.8%), Kerala (−10), Tamil Nadu (−6.1), Tripura (−6%), Mizoram (−4.9%), Lakshadweep (−4.8%), Meghalaya (−1.8%) and Manipur (−0.4%).

Supplementary Table S1 shows the Pearson’s correlation coefficients for state-level coverage of IFA supplementation across beneficiary groups. There is a positive correlation in coverage across various beneficiary groups. However, the strength of the association varies. The performance also varies in terms of end provider of IFA supplements and is correlated with sectoral focus and support. In particular, we find that beneficiary groups with same service provider departments have stronger association than that of beneficiary groups from different facilitating units. There is one-to-one association in coverage among school-going adolescent boys and girls (*r* = 0.96), which implies that when the coverage of IFA supplement improves among the school-going adolescent girls it also seems to improve among school-going boys. Similarly, there is a significant correlation between coverage among adolescents and children. However, there is a weak association between IFA supplementation coverage among PW and lactating mothers (*r* = 0.42). There is a huge difference in IFA supplementation coverage between pregnant and lactating mothers, which also varies by states. Although, at the state level the improvement in IFA tablet or syrup supplementation coverage of one beneficiary group does positively influence the coverage of other groups but the association is even more robust in situation where the IFA supplements supplier and last mile service providers are from the same department (such as all within the health department).

Supplementary Table S2 reports the mean and SD of coverage level across beneficiary groups by states/UTs and by districts for the years 2017–18, 2018–19 and 2019–20. Across the states, in 2019–20, the average coverage ranged from 13.3% (among children 6–59 months) to 78.5% (among PW). The mean coverage for the beneficiary groups is highest among PW (65.9% in 2017–18, 72% in 2018–19 and 78.5% in 2019–20) and lowest among children aged 6–59 months (6% in 2017–18, 8% in 2017–18 and 13.3% in 2019–20). A decline in mean coverage of IFA supplementation is noted among the adolescent boys (29.4% to 23.9% during 2019–20). Among the school going adolescent girls, the mean coverage decreased from 46.4% to 32.6% in 2018–19. However, during 2019–20, there was an increase in coverage from 32.6% to 38.5%. Across the 704 districts, the mean coverage in 2017–18 varied between 7.0% (children aged 6–59 months) and 73.2% (PW). Post-AMB, the coverage across the districts shows improvement for all beneficiary groups except for school-going adolescent girls (reduction from 48.9% to 38.8% during 2019–20). The data show that after a year of AMB implementation, the mean coverage across districts declined for school-going adolescent girls (−19.2%), adolescent boys (−5.8%) and out-of-school girls (−3.7%). The SD values suggest that among the PW, less inter- and intra-state variations are noted compared to other beneficiary groups.

Further, it is also critical to validate the HMIS-based coverage estimates with independent survey-based data. In this regard, Supplementary Figure S2 shows that the direction of increase in coverage as noted in HMIS data is consistent with the movement observed across most of the states between NFHS 2015–16 and NFHS 2019–20. The NFHS estimates suggest that the consumption of IFA tablets during pregnancy (at least for 100 days) has increased from 30.3% in 2015–16 to 44.1% in 2019–21. Also, the correlation of NFHS and HMIS estimates in terms of level of coverage has improved from 0.30 in 2015–16 to 0.48 in 2019–20 (Supplementary Table S10).

Estimates from multilevel regression analysis of coverage of IFA supplementation by beneficiary groups is presented in Table 3. The time period (year) coefficient across beneficiary groups is positive and significant indicating coverage improvements after the implementation of AMB strategy. Higher annual coverage improvements are noted among lactating mothers (4.3 percentage points per year) and PW (4.0 percentage points per year). Coverage improvements among children (6–59 months) is 3.5 percentage points per year and among children (5–9 years) is 3.7% percentage points per year. The slowest improvements are noted among out-of-school girls (1.2 percentage points per year). Districts with larger population tend to have marginally higher coverage. In particular, districts with larger population have noted greater coverage change in case of IFA supplementation among lactating women.

**Table 3.** Multilevel linear regression of IFA supplementation coverage by beneficiary groups, 2017–18 to 2019–20

Beneficiaries	Children (6–59 month)		Children (5–9 years)		Adolescent girls (10–19 years)		Adolescent boys (10–19 years)		Out-of-school girls (10–19 years)		Pregnant women		Lactating mothers	
**Fixed parameters**	**Coef.**	**SE**	**Coef.**	**SE**	**Coef.**	**SE**	**Coef.**	**SE**	**Coef.**	**SE**	**Coef.**	**SE**	**Coef.**	**SE**
Time (2017–18 to 2019–20)	3.459	0.246	3.722	0.491	3.206	0.730	2.855	0.663	1.235	0.799	3.973	0.303	4.341	0.371
Target population (in thousands)	0.015	0.001	0.003	0.000	0.023	0.001	0.016	0.001	0.058	0.004	0.105	0.012	0.584	0.031
Random-effects parameters														
States (SD)	10.3	1.4	10.8	1.5	19.1	2.6	18.5	2.5	19.0	2.5	16.7	2.1	15.9	2.2
District (SD)	5.1	0.3	9.2	0.6	7.6	1.1	9.5	0.8	8.1	1.1	9.3	0.4	13.6	0.5
Residual (SD)	9.1	0.2	16.2	0.3	23.9	0.5	21.6	0.4	24.4	0.5	11.3	0.2	12.7	0.3
VPC (%)														
State/UT level	50%		25%		37%		38%		35%		57%		42%	50%
District level	12%		18%		6%		10%		6%		18%		31%	12%
Residual	38%		57%		57%		52%		58%		26%		27%	38%

Note: All coefficients are significant at 5% level. VPC denotes the variance partition coefficient estimated as variance attributable to a given level divided by total variance.

Source: Authors based on HMIS and AMB dashboard (www.anemiamuktbharat.info).

The random-effects parameters are used to estimate the variance in coverage attributable at state and district levels and across beneficiary groups (Table 3). The VPCs in case of children (6–59 months) suggest that half of the variation in coverage is attributable to differences across states. Only 12% of the coverage variation is because of inter-district differences within states. The coverage variation across school-based supplementation provided to the school-going children and adolescent (5–19 years) or out-of-school adolescents-based supplementation provided to children (6–59 months) and out-of-school girls is largely unexplained and reflects potential role of within-district factors in variations. Nevertheless, one-third of these variations are also attributable to state-level factors. In case of adolescent girls, boys and out-of-school girls (10–19 years), the districts capture small variations in IFA supplementation, while 35% of the variance is related with inter-state variations. Among PW and lactating mothers, the coverage variations are mostly at the state level (57% and 42%, respectively). However, in case of lactating women, substantial variation (31%) is discerned at the district level, suggesting that within states some districts have better coverage than others.

The AMB strategy was formally launched across several states. However, only in seven states (Assam, Bihar, Chhattisgarh, Jharkhand, Madhya Pradesh, Maharashtra and Odisha) trainings and sensitization workshops for the state- and district-level officials were conducted till 2019–20. We utilize this information on programme implementation and present the DD estimates to understand whether conduct of such workshops and trainings across states shares a favourable association with IFA supplementation coverage across beneficiary groups (Table 4). The average district-level coverage in 2017–18 across beneficiary categories were relatively similar for states where such trainings and sensitization workshops were conducted (treatment group) and those without such workshops (comparison group). Only among children (6–59 months and 5–9 years), the coverage in treatment group was significantly lower than the comparison group. In 2019–20, in most of the beneficiary groups, the average district-level IFA supplementation coverage was higher in the treatment states. No significant difference was noted in case of children (5–9 years) and out-of-school adolescent girls. Finally, the DD estimates reveal that conduct of trainings and sensitization workshop is associated with improvements in IFA supplementation coverage for most of the groups (excluding school-going as well as out-of-school adolescent girls).

**Table 4.** Difference-in-difference estimates for impact of training and orientation on IFA supplementation coverage by beneficiary groups, 2017–18 to 2019–20

	Children (6–59 months)	Children (5–9 years)	Adolescent girls (10–19 years)	Adolescent boys (10–19 years)	Out-of-school girls (10–19 years)	Pregnant women	Lactating mothers
2017–18							
Control	7.91	17.61	46.96	35.59	28.56	73.22	38.42
Treated	4.61	9.15	54.26	32.06	30.57	73.43	39.1
Diff (T-C)	−3.29^b^	−8.46^c^	7.3^b^	−3.54	2.01	0.21	0.68
Std. Err.	1.46	2.87	3.55	3.21	3.64	1.79	2.19
2019–20							
Control	13.5	28.21	37.75	36.7	33.34	79.95	44.28
Treated	21.14	27.68	42.66	42.12	33.78	86.43	56.76
Diff (T-C)	7.64^c^	−0.53	4.91^a^	5.43^b^	0.44	6.48^c^	12.49^c^
Std. Err.	1.46	2.46	2.86	2.58	3.04	1.79	2.28
Diff-in-Diff	10.93^c^	7.93^b^	−2.39	8.96^b^	−1.56	6.27^b^	11.81^c^
Std. Err.	2.07	3.78	4.56	4.12	4.74	2.53	3.16

^a^ *P*<.1, ^b^*P*<.05, ^c^*P*<.01.

## Discussion and conclusion

Anaemia is a severe public health problem in India affecting over half of the population in all age groups. Since 1970s, the government of India has devised several programmes like National Nutritional Anemia Control Program, WIFS and NIPI to reduce the prevalence of anaemia in India. Despite widespread efforts, no significant improvement has been noticed in the prevalence of anaemia. In the light of the recent evidence on persistent levels of anaemia prevalence, an intensified NIPI programme referred to as the AMB strategy was launched in 2018. This paper analysed the changes in IFA supplementation coverage since the launch of the AMB strategy.

The salient findings of the study are as follows. First, the IFA supplementation coverage has improved across the beneficiary groups after the implementation of AMB strategy (from 72 million beneficiaries in 2017–18 to 129 million in 2019–20). The AMB index finds that states such as Gujarat, Himachal Pradesh, Andhra Pradesh and Madhya Pradesh have better coverage across beneficiary groups. In terms of improvements between 2017–18 and 2019–20, states such as Himachal Pradesh, Haryana and Bihar register a good performance. Second, across the beneficiary groups, IFA supplementation coverage was highest among PW (90.3%) and lowest among children aged 6–59 months (14.9%). Third, no gender disparities are noted in the coverage of IFA supplementation among school-going girls and boys. This is because both were receiving IFA supplements from the same facilitating units (schools). Prior to the launch of the AMB strategy, the coverage was marginally higher among adolescent girls (22.6%) in comparison to adolescent boys (21.1%). In 2019–20, however, boys have marginally higher coverage of IFA supplementation.

Fourth, coverage improvements across beneficiary groups are correlated. These associations are fairly robust if the source of IFA supplementation (such as health facilities/*Anganwadi* centre (AWC)/schools) is the same for the beneficiary groups. Whereas, there is a weak association in coverage rates of school-going children and lactating mothers. This is because both the groups receive IFA supplement from different service providers—the former from schools (education department) and the latter from health sub-centres (health department). Fifth, bulk of the variations in IFA coverage is attributable to state-specific differences in IFA supplementation coverage. In case of coverage among lactating mothers, the inter-district variations were important, whereas for school-based IFA supplementation, most of the variation was attributable to within-district factors. Finally, the DD estimates reveal that conduct of AMB trainings and sensitization workshops has a favourable role in augmenting IFA supplementation coverage.

AMB as a strategy has features of international best practices that emphasize on community involvement, frontline worker trainings, adequate supplies, deworming, demand generation as well as sufficient financing (Stoltzfus, 2011; Berti *et al.*, 2014; Siekmans *et al.*, 2018). In particular, across low- and middle-income countries, it is argued that programmes should focus on community-based forums with adequate training and supportive supervision of the frontline workers (Kavle and Landry 2018). Provisioning of IFA supplementation during first trimester is an important area where the AMB strategy can further focus with help of frontline workers. It can also address culturally sensitive reasons for delays in registration of pregnancy and other misconceptions around IFA intake and adherence practices through home visits and targeted counselling to address inter-personal, intra-personal or community-level concerns (Shaw *et al.*, 2012; Alam *et al.*, 2015; Sununtnasuk *et al.*, 2016). The AMB strategy although emphasizes on coverage among other beneficiary groups, which is an important requisite for anaemia control programmes in high prevalence low- and middle-income countries (Rashid *et al.*, 2010). The AMB can further improve its scope in terms of epidemiological evaluation of anaemia prevalence by examining iron-deficiency anaemia and other clinical deficiencies to effectively target the IFA supplementation coverage and related micronutrient interventions across beneficiary groups (Pasricha *et al.*, 2013).

The initial phases of the programme had greater focus on IFA supplementation strengthening, and it has led to coverage improvements for various beneficiary groups and across geographies. However, it is important that the improvements in coverage also lead to improvements in levels of anaemia prevalence in the country. Despite the improved coverage, the NFHS 2019–21 indicates increase in anaemia prevalence since NFHS 2015–16, implying the coverage trend is the beginning, not the final step in reducing anaemia. In fact, the association of changes in the coverage level based on HMIS 2017–18 and 2019–20 as well as changes in the anaemia prevalence level among PW based on NFHS 2015–16 and NFHS 2019–21 remains inconclusive (Supplementary Figure S3). Anaemia prevalence among PW has mostly increased in states where the changes in IFA supplementation coverage are smaller but it has reduced for most states where the changes in coverage are larger (more than 10 percentage points). While the observed association between IFA supplementation coverage and anemia prevalence across districts is sensitive to COVID-19 disruptions in service delivery but such patterns also reveal the challenges associated with prevention and control of anaemia. In particular, anaemia has a multifactorial aetiology with a wide range of non-nutritional causes and other biological intricacies that may affect iron absorption tendencies. Besides, increases in coverage alone do not necessarily have the desired impact if the quality aspect of coverage, including frequency of IFA distribution and aspects such as consumption adherence, is not adequately focused upon.

Lack of synergy in programme implementation (both within line department and between line departments) is an important cause of coverage inefficiencies (Avula *et al.*, 2015; Kim *et al.*, 2017; Paul *et al.*, 2018). Especially, it is noticed that when supply chain is highly segmented, convergence efforts with relevant line department require considerable engagement (Wendt *et al.*, 2018). For instance, effective coverage among school children requires extensive coordination with Department of Education (Bhatia *et al.*, 2019; Kapil *et al.*, 2019a). Likewise, for children under-five (as well as for out-of-school girls) who are provided IFA supplementation through frontline *Anganwadi* workers, it is a prerequisite to coordinate with the AWCs under the Integrated Child Development Services programme of the Ministry of Women and Child Development. Given such intricacies, proactive planning is necessary for an efficient and functional IFA supply chain. The current state-of-affairs, however, is far from desirable. An elementary concern is apparent in the form of poor cooperation in regular reporting of stocks and coverage across inter-departmental operations (such as, schools or AWCs). The system is found to suffer not only from logistics and resources perspective but also has incentive problems (Kapil *et al.*, 2019a; Bhatia *et al.*, 2020). Systemic motivation and capacity building may perhaps have limited merit if targets are too ambitious to be reached within a stipulated time frame. Moreover, increased coverage has to be translated into improved adherence to make an impact on prevalence levels. For this purpose, the frequency of IFA distribution across beneficiary groups also needs rigorous monitoring. For instance, distribution of IFA supplements for PW (180 tablets) is among best implemented practices, but distribution efficacy for young children or even school-going or out-of-school children is sub-optimal (Kapil *et al.*, 2019a). Irregularities in distribution would invariably lead to poor coverage and may not necessarily impact anaemia prevalence (Berry *et al.*, 2020). With distribution as a concern, food fortification strategies are increasingly debated as an alternative to address iron deficiency. Although the advantage of food fortification in terms of wider coverage is apparent, there is mixed evidence on effectiveness of such measures on prevalence levels (Martorell *et al.*, 2015; Swaminathan *et al.*, 2019).

Progress notwithstanding, IFA supplementation coverage is a complex issue with problems associated with IFA supply chain management under national health programmes (Wendt *et al.*, 2018; Kapil *et al.*, 2019a; Bhatia *et al.*, 2020). The supply chain management involves dedicated managerial tasks of demand forecasting, procurement (including timely delivery by manufacturers), inventory and warehousing provisions, transportation, drugs distribution (including last mile delivery) and stock reporting. It is increasingly recognized that supply chain bottlenecks adversely affect last mile delivery and IFA supplementation coverage (Diamond-Smith *et al.*, 2016; Varghese *et al.*, 2019). For instance, Wendt *et al.* (2018) examined the contributing barriers to insufficient IFA supply in Bihar and noted major gaps in approach towards process documentation, forecasting drug demand, procurement policy and management of stock requests. Nevertheless, some states (such as Tamil Nadu and Madhya Pradesh) have more streamlined processes for supply chain management including procurement and last mile delivery of IFA supplements (Singh *et al.*, 2012; Chakma *et al.*, 2013).

Rigorous monitoring of AMB strategy is an important area to improve ground-level performance and dismantle implementation barriers (Paul *et al.*, 2018; Kapil *et al.*, 2019a). Improvements in data reporting and data quality of AMB indicators under the HMIS system can establish a robust mechanism to measure the progress of programme. Promoting meso-level monitoring and evaluations can improve the delivery of health services for all the target groups (Hipgrave *et al.*, 2014). Simultaneously, there should be a focus on demand generation across target groups such that supplies can be improved with repeated demands from line departments, particularly from schools and AWCs. Similarly, regular replenishment of drug kits of ANM and ASHA workers is also necessary for effective last mile delivery and continuous monitoring through monthly review meetings at district or project/block levels (Varghese *et al.*, 2019). A workable convergence action plan is also relevant for enhancing the coverage across beneficiary groups from inter-sectoral categories viz. schools and AWCs (Khapre *et al.*, 2020).

The DD analysis is based on a small sample but nevertheless it suggests that AMB trainings and sensitization workshops are associated with improvements in coverage for most of the beneficiary groups. Partly, this could be attributable to improvements in reporting of the coverage as officials are more likely to focus on achievements through rectified reporting. Besides, such events also lead to increased sensitivity within the health system and allied sectors to focus on the IFA supplementation efforts. A concerted focus on training and capacity building of AMB officials and frontline workers across line departments can lead to substantial changes in IFA supplementation coverage across beneficiary groups (Car *et al.*, 2018). For instance, there is poor inter-departmental data sharing of IFA supplementation coverage across schools and AWCs. Nevertheless, such suggestive evidence calls for further research necessary to understand the role and limitations of line departments to work in convergence to achieve the programme objectives.

The analysis, however, has a few limitations. The coverage estimates are based on the HMIS data that rely on the state-level apparatus on data reporting and accuracy. Also, there are time lags in reporting of data. The analysis focuses on the coverage of IFA supplementation, which does not necessarily imply consumption of IFA, particularly among PW and lactating mothers. The data reporting during COVID-19 pandemic were disrupted, and hence, the completeness of coverage data for the 2019–20 is an important concern. Also, the change in coverage although can be associated with AMB strategy as well as activities such as training and sensitization workshops, but further information and analysis will be necessary to draw any causal relationships. Despite these shortcomings, it is apparent that AMB is an important chapter in IFA supplementation coverage in India, and it is critical that AMB strategy is effectively implemented in the post-pandemic phase, both in principle and in practice.

To conclude, it is encouraging to notice improvements in AMB coverage across beneficiary groups, but the pace of improvement has to be sustained to make a dent on anaemia in India. However, there are a number of challenges before the programme. The AMB strategy will also need concerted efforts to overcome the COVID-19-related disruptions and resume activities for sustained coverage and outreach. This includes rejuvenating the efforts for demand generation for IFA supplements across beneficiary groups. The various line departments (Health Department, School Education Department and the *Anganwadi* services) should display greater convergence and coordination to ensure timely distribution, coverage and reporting. Equal attention is also necessary to ensure regular supplies to sustain coverage levels. Besides, it is important that the HMIS should be made more dynamic to accommodate new indicators pertaining to the AMB strategy. Training and sensitization of AMB officials can further improve reporting of stocks and coverage statistics from other line departments responsible for distribution across schools and AWCs. The programme also needs to streamline last mile delivery. Improved testing, tracking and treatment of anaemic patients are also an area that requires concerted engagements. The AMB had also undertaken some important modification in dosage volume of IFA supplements and reduced elemental iron content from 100-mg to 60-mg formulations. But the new dosage-based supplements have to be manufactured, procured and distributed. It should be also accompanied with awareness campaigns on how it can improve side effects that were associated with heavy dosages. Finally, even after able implementation of the IFA supplementation strategies, it is probable that the levels of anaemia prevalence may continue to be affected by non-nutritional causes including malaria, haemoglobinopathies and fluorosis, thereby rendering IFA supplementation only partly effective. A system-wide effective implementation of the all-encompassing determinants-based AB strategy is necessary to reduce the prevalence of anaemia in India.

## Supplementary Material

czac015_Supp

## Data Availability

The data underlying this article will be shared on reasonable request to the corresponding author.
