# Supplementary material for: Coverage of iron and folic acid supplementation in India: progress under the Anemia Mukt Bharat strategy 2017–20
Source: Health Policy Plan. 2022 Feb 28;37(5):597–606. doi: 10.1093/heapol/czac015 (PMC9113188; doi:10.1093/heapol/czac015)
Supplement: czac015_Supp [file czac015_supp.zip › IFA_Supplementation_Coverage_Supplementary_Tables_Figures_Nov29_2021.docx]

**Supplementary Tables and Figures**

**Supplementary Table S1**: District-level correlation coefficient for IFA supplementation coverage across beneficiary groups, India 2019-20

| **2019-20** | Pregnant women | Lactating mother | Children  (6-59 months) | Adolescent girls | Adolescent boys | Out-of-school girls | Children  (5-9) years |
| --- | --- | --- | --- | --- | --- | --- | --- |
| Pregnant women | 1.00 |  |  |  |  |  |  |
| Lactating mother | 0.42* | 1.00 |  |  |  |  |  |
| Children (6-59 months) | 0.22* | 0.35* | 1.00 |  |  |  |  |
| Adolescent girls | 0.30* | 0.21* | 0.23* | 1.00 |  |  |  |
| Adolescent boys | 0.28* | 0.17* | 0.22* | 0.96* | 1.00 |  |  |
| Out-of-school girls | 0.22* | 0.33* | 0.38* | 0.36* | 0.36* | 1.00 |  |
| Children (5-9) years | 0.28* | 0.21* | 0.25* | 0.47* | 0.49* | 0.14* | 1.00 |

*Source*: Authors based on HMIS and AMB dashboard ([www.anemiamuktbharat.info](http://www.anemiamuktbharat.info))

**Supplementary Table S2**: Mean and standard deviation of IFA supplementation coverage across beneficiary groups by a) States/UTs and b) Districts, India 2017-18 to 2019-20

| **States/UTs** | 2017-18 | | 2018-19 | | 2019-20 | |
| --- | --- | --- | --- | --- | --- | --- |
|  | Mean | SD | Mean | SD | Mean | SD |
| Children (6-59 months) | 6.0 | 10.4 | 8.0 | 14.9 | 13.3 | 20.2 |
| Children (5-9 Years) | 11.2 | 17.0 | 14.1 | 24.5 | 39.1 | 28.6 |
| School going adolescent girls | 46.4 | 39.2 | 32.6 | 29.3 | 38.5 | 28.6 |
| Adolescent boys | 27.4 | 24.9 | 29.4 | 27.8 | 23.9 | 26.7 |
| Out of school adolescent girls | 17.7 | 24.9 | 21.2 | 28.8 | 33.6 | 32.4 |
| Pregnant women | 65.9 | 24.0 | 72.0 | 24.5 | 78.5 | 19.9 |
| Lactating mother | 41.0 | 23.6 | 49.6 | 24.6 | 53.9 | 24.1 |
| **Districts** | 2017-18 | | 2018-19 | | 2019-20 | |
|  | Mean | SD | Mean | SD | Mean | SD |
| Children (6-59 months) | 6.9 | 14.0 | 9.1 | 16.5 | 15.4 | 20.0 |
| Children (5-9 Years) | 15.4 | 26.2 | 16.7 | 28.1 | 28.4 | 31.2 |
| School going adolescent girls | 48.8 | 39.0 | 29.6 | 32.5 | 38.8 | 29.7 |
| Adolescent boys | 34.7 | 31.5 | 28.9 | 32.1 | 38.1 | 29.9 |
| Out of school adolescent girls | 29.2 | 35.6 | 25.5 | 34.5 | 33.1 | 32.9 |
| Pregnant women | 73.1 | 23.6 | 75.8 | 23.8 | 81.5 | 18.4 |
| Lactating mother | 38.6 | 26.2 | 44.2 | 26.6 | 48.2 | 25.6 |

*Source*: Authors based on HMIS and AMB dashboard ([www.anemiamuktbharat.info](http://www.anemiamuktbharat.info))

**Supplementary Table S3**: Percentage of IFA supplementation coverage among children 6-59 months across States/UTs, India 2017-18 to 2019-20

| Sates | 2017-18 | 2019-20 | Change(2017-18-2019-20) |
| --- | --- | --- | --- |
| Andaman & Nicobar Islands | 17.2 | 95.0 | 77.8 |
| Andhra Pradesh | 31.1 | 3.5 | -27.6 |
| Arunachal Pradesh | 0.0 | 0.0 | 0.0 |
| Assam | 13.4 | 34.9 | 21.5 |
| Bihar | 0.0 | 12.2 | 12.2 |
| Chandigarh | 1.3 | 0.1 | -1.2 |
| Chhattisgarh | 4.2 | 7.4 | 3.2 |
| Dadra & Nagar Haveli | 14.8 | 26.9 | 12.1 |
| Daman & Diu | 1.5 | 1.8 | 0.3 |
| Goa | 0.0 | 0.0 | 0.0 |
| Gujarat | 18.6 | 21.3 | 2.7 |
| Haryana | 3.5 | 44.9 | 41.4 |
| Himachal Pradesh | 0.3 | 23.6 | 23.3 |
| Jammu & Kashmir | 2.1 | 0.7 | -1.4 |
| Jharkhand | 0.0 | 2.2 | 2.2 |
| Karnataka | 3.9 | 1.3 | -2.6 |
| Kerala | 0.2 | 0.4 | 0.2 |
| Lakshadweep | 0.0 | 0.0 | 0.0 |
| Madhya Pradesh | 12.4 | 26.0 | 13.6 |
| Maharashtra | 0.1 | 39.3 | 39.2 |
| Manipur | 0.6 | 0.9 | 0.3 |
| Meghalaya | 0.3 | 0.6 | 0.3 |
| Mizoram | 0.4 | 0.0 | -0.4 |
| Nagaland | 0.2 | 0.0 | -0.2 |
| NCT of Delhi | 0.2 | 0.1 | -0.1 |
| Orissa | 12.2 | 27.4 | 15.2 |
| Puducherry | 9.6 | 6.8 | -2.8 |
| Punjab | 11.5 | 6.6 | -4.9 |
| Rajasthan | 1.1 | 17.5 | 16.4 |
| Sikkim | 0.9 | 7.8 | 6.9 |
| Tamil Nadu | 2.4 | 6.8 | 4.4 |
| Telangana | 0.1 | 7.6 | 7.5 |
| Tripura | 0.0 | 0.1 | 0.1 |
| Uttar Pradesh | 0.4 | 0.5 | 0.1 |
| Uttarakhand | 0.4 | 0.1 | -0.3 |
| West Bengal | 49.3 | 53.7 | 4.4 |
| India | 6.6 | 14.9 | 8.3 |

*Source*: Authors based on HMIS and AMB dashboard ([www.anemiamuktbharat.info](http://www.anemiamuktbharat.info))

**Supplementary Table S4**: Percentage of IFA supplementation coverage among WIFS JUNIOR (5-9 years) across States/UTs, India 2017-18 to 2019-20

| Sates | 2017-18 | 2019-20 | Change  (2017-18-2019-20) |
| --- | --- | --- | --- |
| Andaman & Nicobar Islands | NA | 95.0 | NA |
| Andhra Pradesh | 37.7 | 32.9 | -4.8 |
| Arunachal Pradesh | 0.3 | 2.6 | 2.3 |
| Assam | 13.1 | 49.1 | 36.0 |
| Bihar | 0.0 | 15.1 | 15.1 |
| Chandigarh | NA | 0.0 | NA |
| Chhattisgarh | NA | 0.6 | 0.6 |
| Dadra & Nagar Haveli | 1.1 | 56.0 | 54.9 |
| Daman & Diu | 0.6 | 35.2 | 34.6 |
| Goa | NA | 0.0 | NA |
| Gujarat | 27.2 | 32.5 | 5.3 |
| Haryana | 0.0 | 2.0 | 2.0 |
| Himachal Pradesh | 0.0 | 36.5 | 36.5 |
| Jammu & Kashmir | 2.3 | 0.3 | -2.0 |
| Jharkhand | 0.0 | 11.9 | 11.9 |
| Karnataka | 15.1 | 39.5 | 24.4 |
| Kerala | 0.1 | 0.0 | -0.1 |
| Lakshadweep | 0.0 | 0.0 | 0.0 |
| Madhya Pradesh | 34.5 | 70.4 | 35.9 |
| Maharashtra | 10.9 | 56.8 | 45.9 |
| Manipur | 6.5 | 2.7 | -3.8 |
| Meghalaya | 0.0 | 0.2 | 0.2 |
| Mizoram | 13.2 | 0.2 | -13.0 |
| Nagaland | 0.2 | 0.0 | -0.2 |
| NCT of Delhi | 0.0 | 0.0 | 0.0 |
| Orissa | 18.4 | 37.9 | 19.5 |
| Puducherry | 78.8 | 79.9 | 1.1 |
| Punjab | 36.5 | 28.1 | -8.4 |
| Rajasthan | 8.0 | 7.2 | -0.8 |
| Sikkim | 26.1 | 1.9 | -24.2 |
| Tamil Nadu | 10.8 | 59.0 | 48.2 |
| Telangana | 7.0 | 9.3 | 2.3 |
| Tripura | 3.4 | 0.9 | -2.5 |
| Uttar Pradesh | 5.7 | 53.4 | 47.7 |
| Uttarakhand | 0.0 | 18.9 | 18.9 |
| West Bengal | 0.7 | 23.4 | 22.7 |
| India | 8.0 | 30.2 | 22.2 |

*Source*: Authors based on HMIS and AMB dashboard ([www.anemiamuktbharat.info](http://www.anemiamuktbharat.info))

**Supplementary Table S5**: Percentage of IFA supplementation coverage among adolescent school going girls (10-19) across States/UTs, India 2017-18 to 2019-20

| Sates | 2017-18 | 2019-20 | Change  (2017-18-2019-20) |
| --- | --- | --- | --- |
| Andaman & Nicobar Islands | NA | 95.0 | NA |
| Andhra Pradesh | 95.0 | 77.4 | -17.6 |
| Arunachal Pradesh | 1.0 | 7.1 | 6.1 |
| Assam | 95.0 | 49.3 | -45.7 |
| Bihar | 0.0 | 44.2 | 44.2 |
| Chandigarh | 95.0 | 7.2 | -87.8 |
| Chhattisgarh | 95.0 | 54.9 | -40.1 |
| Dadra & Nagar Haveli | 49.9 | 57.8 | 7.9 |
| Daman & Diu | 22.4 | 35.9 | 13.5 |
| Goa | 18.0 | 95.0 | 77.0 |
| Gujarat | 30.1 | 46.1 | 16.0 |
| Haryana | 95.0 | 77.6 | -17.4 |
| Himachal Pradesh | 13.6 | 65.0 | 51.4 |
| Jammu & Kashmir | 5.2 | 0.4 | -4.8 |
| Jharkhand | 22.6 | 40.1 | 17.5 |
| Karnataka | 95.0 | 61.1 | -33.9 |
| Kerala | 95.0 | 0.0 | -95.0 |
| Lakshadweep | 0.0 | 0.0 | 0.0 |
| Madhya Pradesh | 95.0 | 64.1 | -30.9 |
| Maharashtra | 95.0 | 42.2 | -52.8 |
| Manipur | 23.4 | 5.8 | -17.6 |
| Meghalaya | 37.3 | 17.9 | -19.4 |
| Mizoram | 95.0 | 26.1 | -68.9 |
| Nagaland | 2.1 | 9.9 | 7.8 |
| NCT of Delhi | NA | 3.6 | NA |
| Orissa | 18.2 | 38.3 | 20.1 |
| Puducherry | 71.5 | 95.0 | 23.5 |
| Punjab | 68.3 | 34.6 | -33.7 |
| Rajasthan | 17.0 | 9.6 | -7.4 |
| Sikkim | 95.0 | 61.1 | -33.9 |
| Tamil Nadu | 80.6 | 50.3 | -30.3 |
| Telangana | 7.8 | 30.9 | 23.1 |
| Tripura | 26.2 | 2.0 | -24.2 |
| Uttar Pradesh | 4.7 | 46.4 | 41.7 |
| Uttarakhand | 1.7 | 28.5 | 26.8 |
| West Bengal | 11.0 | 26.7 | 15.7 |
| India | 22.6 | 39.7 | 17.1 |

*Source*: Authors based on HMIS and AMB dashboard ([www.anemiamuktbharat.info](http://www.anemiamuktbharat.info))

**Supplementary Table S6**: Percentage of IFA supplementation coverage among adolescent school going boys (10-19) across States/UTs, India 2017-18 to 2019-20

| Sates | 2017-18 | 2019-20 | Change  (2017-18-2019-20) |
| --- | --- | --- | --- |
| Andaman & Nicobar Islands | NA | 95.0 | NA |
| Andhra Pradesh | 44.0 | 84.2 | 40.2 |
| Arunachal Pradesh | 2.0 | 5.9 | 3.9 |
| Assam | 27.3 | 45.3 | 18.0 |
| Bihar | 0.0 | 44.9 | 44.9 |
| Chandigarh | 56.6 | 7.4 | -49.2 |
| Chhattisgarh | 14.1 | 56.0 | 41.9 |
| Dadra & Nagar Haveli | 12.0 | 51.5 | 39.5 |
| Daman & Diu | 60.7 | 35.8 | -24.9 |
| Goa | 35.1 | 95.0 | 59.9 |
| Gujarat | 22.6 | 75.1 | 52.5 |
| Haryana | 11.7 | 79.8 | 68.1 |
| Himachal Pradesh | 12.2 | 65.8 | 53.6 |
| Jammu & Kashmir | 2.0 | 0.4 | -1.6 |
| Jharkhand | 27.8 | 34.5 | 6.7 |
| Karnataka | 28.0 | 63.8 | 35.8 |
| Kerala | 19.3 | 1.5 | -17.8 |
| Lakshadweep | 0.0 | 0.0 | 0.0 |
| Madhya Pradesh | 33.1 | 67.3 | 34.2 |
| Maharashtra | 46.3 | 44.0 | -2.3 |
| Manipur | 2.9 | 5.1 | 2.2 |
| Meghalaya | 29.1 | 15.4 | -13.7 |
| Mizoram | 49.3 | 25.6 | -23.7 |
| Nagaland | 7.2 | 9.7 | 2.5 |
| NCT of Delhi | NA | 3.0 | NA |
| Orissa | 17.5 | 37.5 | 20.0 |
| Puducherry | 64.4 | 65.0 | 0.6 |
| Punjab | 65.9 | 32.7 | -33.2 |
| Rajasthan | 14.4 | 8.8 | -5.6 |
| Sikkim | 95.0 | 62.9 | -32.1 |
| Tamil Nadu | 81.9 | 45.6 | -36.3 |
| Telangana | 7.9 | 26.7 | 18.8 |
| Tripura | 25.8 | 1.9 | -23.9 |
| Uttar Pradesh | 4.7 | 42.0 | 37.3 |
| Uttarakhand | 1.6 | 25.0 | 23.4 |
| West Bengal | 10.1 | 25.5 | 15.4 |
| India | 21.1 | 41.6 | 20.5 |

*Source*: Authors based on HMIS and AMB dashboard ([www.anemiamuktbharat.info](http://www.anemiamuktbharat.info))

**Supplementary Table S7**: Percentage of IFA supplementation coverage among out-of-school girls (10-19) across States/UTs, India 2017-18 to 2019-20

| States | 2017-18 | 2019-20 | Change  (2017-18-2019-20) |
| --- | --- | --- | --- |
| Andaman & Nicobar Islands | NA | 42.6 | NA |
| Andhra Pradesh | 64.0 | 95.0 | 31.0 |
| Arunachal Pradesh | 0.0 | NA | NA |
| Assam | 29.8 | 47.0 | 17.2 |
| Bihar | 0.0 | 18.8 | 18.8 |
| Chandigarh | 0.6 | 15.8 | 15.2 |
| Chhattisgarh | 8.5 | 54.0 | 45.5 |
| Dadra & Nagar Haveli | 0.6 | 0.0 | -0.6 |
| Daman & Diu | 49.8 | 0.0 | -49.8 |
| Goa | 0.2 | 61.0 | 60.8 |
| Gujarat | 95.0 | 95.0 | 0.0 |
| Haryana | 8.5 | 82.5 | 74.0 |
| Himachal Pradesh | 0.1 | 95.0 | 94.9 |
| Jammu & Kashmir | 1.7 | 0.4 | -1.3 |
| Jharkhand | 13.1 | 22.9 | 9.8 |
| Karnataka | 15.7 | 14.4 | -1.3 |
| Kerala | 0.0 | 0.1 | 0.1 |
| Lakshadweep | 0.4 | 0.0 | -0.4 |
| Madhya Pradesh | 5.5 | 49.2 | 43.7 |
| Maharashtra | 49.2 | 56.9 | 7.7 |
| Manipur | 3.9 | 8.5 | 4.6 |
| Meghalaya | 6.9 | 4.3 | -2.6 |
| Mizoram | 34.0 | 69.0 | 35.0 |
| Nagaland | 0.2 | 3.7 | 3.5 |
| NCT of Delhi | NA | 1.1 | NA |
| Orissa | 11.4 | 35.2 | 23.8 |
| Puducherry | 74.7 | 95.0 | 20.3 |
| Punjab | 18.9 | 8.8 | -10.1 |
| Rajasthan | 22.1 | 25.0 | 2.9 |
| Sikkim | 3.2 | 0.1 | -3.1 |
| Tamil Nadu | 60.2 | 18.7 | -41.5 |
| Telangana | 10.3 | 25.6 | 15.3 |
| Tripura | 0.3 | 0.5 | 0.2 |
| Uttar Pradesh | 2.6 | 16.5 | 13.9 |
| Uttarakhand | 0.0 | 47.1 | 47.1 |
| West Bengal | 8.8 | 65.0 | 56.2 |
| India | 6.4 | 22.7 | 16.3 |

*Source*: Authors based on HMIS and AMB dashboard ([www.anemiamuktbharat.info](http://www.anemiamuktbharat.info))

**Supplementary Table S8**: Percentage of IFA supplementation coverage among pregnant women (PW)across States/UTs, India 2017-18 to 2019-20

| States | 2017-18 | 2019-20 | Change  (2017-18-2019-20) |
| --- | --- | --- | --- |
| Andaman & Nicobar Islands | 59.7 | 95.0 | 35.3 |
| Andhra Pradesh | 95.1 | 95.0 | 0.0 |
| Arunachal Pradesh | 38.8 | 58.1 | 19.3 |
| Assam | 93.2 | 95.0 | 1.8 |
| Bihar | 51.4 | 77.9 | 26.5 |
| Chandigarh | 72.3 | 95.0 | 22.7 |
| Chhattisgarh | 95.0 | 95.0 | 0.0 |
| Dadra & Nagar Haveli | 74.3 | 86.7 | 12.4 |
| Daman & Diu | 68.0 | 95.0 | 27.0 |
| Goa | 62.6 | 75.1 | 12.5 |
| Gujarat | 95.0 | 95.0 | 0.0 |
| Haryana | 72.2 | 72.8 | 0.6 |
| Himachal Pradesh | 58.2 | 84.8 | 26.6 |
| Jammu & Kashmir | 53.8 | 44.4 | -9.4 |
| Jharkhand | 69.2 | 79.7 | 10.5 |
| Karnataka | 95.0 | 95.0 | 0.0 |
| Kerala | 69.5 | 95.0 | 25.5 |
| Lakshadweep | 94.3 | 95.0 | 0.7 |
| Madhya Pradesh | 92.9 | 93.1 | 0.2 |
| Maharashtra | 91.7 | 95.0 | 3.3 |
| Manipur | 45.9 | 49.3 | 3.4 |
| Meghalaya | 34.4 | 42.6 | 8.2 |
| Mizoram | 24.1 | 50.7 | 26.6 |
| Nagaland | 9.1 | 19.7 | 10.6 |
| NCT of Delhi | 44.9 | 95.0 | 50.1 |
| Orissa | 95.0 | 86.4 | -8.6 |
| Puducherry | 24.5 | 64.6 | 40.1 |
| Punjab | 78.0 | 71.5 | -6.5 |
| Rajasthan | 50.8 | 76.1 | 25.3 |
| Sikkim | 66.7 | 63.8 | -2.9 |
| Tamil Nadu | 89.9 | 92.4 | 2.5 |
| Telangana | 86.0 | 95.0 | 9.0 |
| Tripura | 38.8 | 46.8 | 8.0 |
| Uttar Pradesh | 85.3 | 95.0 | 9.7 |
| Uttarakhand | 46.7 | 74.3 | 27.6 |
| West Bengal | 50.0 | 85.0 | 35.0 |
| India | 77.7 | 90.3 | 12.6 |

*Source*: Authors based on HMIS and AMB dashboard ([www.anemiamuktbharat.info](http://www.anemiamuktbharat.info))

**Supplementary Table S9**: Percentage of IFA supplementation coverage among lactating mothers across States/UTs, India 2017-18 to 2019-20

| States | 2017-18 | 2019-20 | Change  (2017-18-2019-20) |
| --- | --- | --- | --- |
| Andaman & Nicobar Islands | 41.0 | 88.3 | 47.3 |
| Andhra Pradesh | 56.3 | 41.1 | -15.2 |
| Arunachal Pradesh | 32.7 | 37.1 | 4.4 |
| Assam | 47.4 | 85.2 | 37.8 |
| Bihar | 16.8 | 33.4 | 16.6 |
| Chandigarh | 95.0 | 95.0 | 0.0 |
| Chhattisgarh | 51.5 | 55.5 | 4.0 |
| Dadra & Nagar Haveli | 38.6 | 86.7 | 48.1 |
| Daman & Diu | 32.3 | 50.6 | 18.3 |
| Goa | 72.7 | 89.5 | 16.8 |
| Gujarat | 68.8 | 76.5 | 7.7 |
| Haryana | 14.6 | 38.0 | 23.4 |
| Himachal Pradesh | 39.3 | 66.2 | 26.9 |
| Jammu & Kashmir | 47.0 | 77.4 | 30.4 |
| Jharkhand | 37.6 | 54.3 | 16.7 |
| Karnataka | 43.0 | 70.1 | 27.1 |
| Kerala | 19.6 | 36.9 | 17.3 |
| Lakshadweep | 58.7 | 24.9 | -33.8 |
| Madhya Pradesh | 53.5 | 49.9 | -3.6 |
| Maharashtra | 51.9 | 73.5 | 21.6 |
| Manipur | 12.1 | 20.0 | 7.9 |
| Meghalaya | 23.9 | 38.4 | 14.5 |
| Mizoram | 8.2 | 18.1 | 9.9 |
| Nagaland | 5.7 | 12.6 | 6.9 |
| NCT of Delhi | 44.8 | 49.4 | 4.6 |
| Orissa | 34.2 | 49.0 | 14.8 |
| Puducherry | 41.5 | 90.3 | 48.8 |
| Punjab | 49.0 | 49.5 | 0.5 |
| Rajasthan | 15.9 | 27.9 | 12.0 |
| Sikkim | 91.4 | 48.0 | -43.4 |
| Tamil Nadu | 29.5 | 40.1 | 10.6 |
| Telangana | 94.2 | 95.0 | 0.8 |
| Tripura | 17.1 | 17.2 | 0.1 |
| Uttar Pradesh | 17.1 | 38.2 | 21.1 |
| Uttarakhand | 14.0 | 52.9 | 38.9 |
| West Bengal | 58.9 | 64.3 | 5.4 |
| India | 34.4 | 49.0 | 15.5 |

*Source*: Authors based on HMIS and AMB dashboard ([www.anemiamuktbharat.info](http://www.anemiamuktbharat.info))

**Supplementary Table S10**: Coverage level of IFA tablets for pregnant women based on NFHS and HMIS data and the changes between the two selected years, India

| States | NFHS 2019-21 | NFHS 2015-16 | Change | HMIS 2017-18 | HMIS 2019-20 | Change |
| --- | --- | --- | --- | --- | --- | --- |
| A & N Islands | 52.1 | 33.7 | 18.4 | 59.7 | 95 | 35.3 |
| Andhra Pradesh | 41.1 | 30.6 | 10.5 | 95.1 | 95 | 0 |
| Arunachal Pradesh | 8.6 | 2.3 | 6.3 | 38.8 | 58.1 | 19.3 |
| Assam | 18.5 | 5.6 | 12.9 | 93.2 | 95 | 1.8 |
| Bihar | 9.3 | 2.3 | 7 | 51.4 | 77.9 | 26.5 |
| Chandigarh | 64.5 | 29.8 | 34.7 | 72.3 | 95 | 22.7 |
| Chhattisgarh | 26.3 | 9.5 | 16.8 | 95 | 95 | 0 |
| DND & DD | 36.2 | 26.2 | 10 | 74.3 | 86.7 | 12.4 |
| Goa | 65 | 52.8 | 12.2 | 62.6 | 75.1 | 12.5 |
| Gujarat | 43.2 | 18.5 | 24.7 | 95 | 95 | 0 |
| Haryana | 32 | 14.3 | 17.7 | 72.2 | 72.8 | 0.6 |
| Himachal Pradesh | 43 | 22.7 | 20.3 | 58.2 | 84.8 | 26.6 |
| Jammu & Kashmir | 15.9 | 16.7 | -0.8 | 53.8 | 44.4 | -9.4 |
| Jharkhand | 14.9 | 4.2 | 10.7 | 69.2 | 79.7 | 10.5 |
| Karnataka | 26.7 | 32.6 | -5.9 | 95 | 95 | 0 |
| Kerala | 67 | 47.4 | 19.6 | 69.5 | 95 | 25.5 |
| Lakshdweep | 61.7 | 59.1 | 2.6 | 94.3 | 95 | 0.7 |
| Madhya Pradesh | 31.8 | 9.2 | 22.6 | 92.9 | 93.1 | 0.2 |
| Maharashtra | 30.9 | 28 | 2.9 | 91.7 | 95 | 3.3 |
| Manipur | 30.3 | 16.3 | 14 | 45.9 | 49.3 | 3.4 |
| Meghalaya | 20.6 | 13 | 7.6 | 34.4 | 42.6 | 8.2 |
| Mizoram | 10.5 | 2.7 | 7.8 | 24.1 | 50.7 | 26.6 |
| Nagaland | 4.1 | 2.2 | 1.9 | 9.1 | 19.7 | 10.6 |
| NCT Delhi | 49 | 29.9 | 19.1 | 44.9 | 95 | 50.1 |
| Odisha | 34.4 | 4.2 | 30.2 | 95 | 86.4 | -8.6 |
| Puducherry | 64.9 | 36.3 | 28.6 | 24.5 | 64.6 | 40.1 |
| Punjab | 40.5 | 19.9 | 20.6 | 78 | 71.5 | -6.5 |
| Rajasthan | 14.4 | 6 | 8.4 | 50.8 | 76.1 | 25.3 |
| Sikkim | 31.5 | 26.8 | 4.7 | 66.7 | 63.8 | -2.9 |
| Tamil Nadu | 63.1 | 40.1 | 23 | 89.9 | 92.4 | 2.5 |
| Telangana | 34.4 | 28.8 | 5.6 | 86 | 95 | 9 |
| Tripura | 8.9 | 1.6 | 7.3 | 38.8 | 46.8 | 8 |
| Uttar Pradesh | 9.7 | 3.9 | 5.8 | 85.3 | 95 | 9.7 |
| Uttarakhand | 25 | 7.2 | 17.8 | 46.7 | 74.3 | 27.6 |
| West Bengal | 30.8 | 6 | 24.8 | 50 | 85 | 35 |

**Supplementary Table S11**: Prevalence of anemia among pregnant women and children (6-59 months) as per NFHS 2019-21 and NFHS 2015-16, India

| States | Pregnant Women | | | Children (6-59 months) | | |
| --- | --- | --- | --- | --- | --- | --- |
|  | NFHS 2019-21 | NFHS 2015-16 | Change | NFHS 2019-21 | NFHS 2015-16 | Change |
| Andaman & Nicobar Islands | 53.7 | 61.4 | -7.7 | 40 | 49 | -9 |
| Andhra Pradesh | 53.7 | 52.9 | 0.8 | 63.2 | 58.6 | 4.6 |
| Arunachal Pradesh | 27.9 | 37.8 | -9.9 | 56.6 | 54.2 | 2.4 |
| Assam | 54.2 | 44.8 | 9.4 | 68.4 | 35.7 | 32.7 |
| Bihar | 63.1 | 58.3 | 4.8 | 69.4 | 63.5 | 5.9 |
| Chandigarh |  |  |  | 54.6 | 73.1 | -18.5 |
| Chhattisgarh | 51.8 | 41.5 | 10.3 | 67.2 | 41.6 | 25.6 |
| DND & DD | 60.7 | 62.3 | -1.6 | 75.8 | 82 | -6.2 |
| Goa | 41 | 26.7 | 14.3 | 53.2 | 48.3 | 4.9 |
| Gujarat | 62.6 | 51.3 | 11.3 | 79.7 | 62.6 | 17.1 |
| Haryana | 56.5 | 55 | 1.5 | 70.4 | 71.7 | -1.3 |
| Himachal Pradesh | 42.2 | 50.4 | -8.2 | 55.4 | 53.7 | 1.7 |
| Jammu & Kashmir | 44.1 | 46.9 | -2.8 | 72.7 | 53.8 | 18.9 |
| Jharkhand | 56.8 | 62.6 | -5.8 | 67.5 | 69.9 | -2.4 |
| Karnataka | 45.7 | 45.4 | 0.3 | 65.5 | 60.9 | 4.6 |
| Kerala | 31.4 | 22.6 | 8.8 | 39.4 | 35.7 | 3.7 |
| Lakshdweep | 20.9 | 39 | -18.1 | 43.1 | 53.6 | -10.5 |
| Madhya Pradesh | 52.9 | 54.6 | -1.7 | 72.7 | 68.9 | 3.8 |
| Maharashtra | 45.7 | 49.3 | -3.6 | 68.9 | 53.8 | 15.1 |
| Manipur | 32.4 | 26 | 6.4 | 42.8 | 23.9 | 18.9 |
| Meghalaya | 45 | 53.3 | -8.3 | 45.1 | 48 | -2.9 |
| Mizoram | 34 | 27 | 7 | 46.4 | 19.3 | 27.1 |
| Nagaland | 22.2 | 32.7 | -10.5 | 42.7 | 26.4 | 16.3 |
| NCT Delhi | 42.2 | 46.1 | -3.9 | 69.2 | 59.7 | 9.5 |
| Odisha | 61.8 | 47.6 | 14.2 | 64.2 | 44.6 | 19.6 |
| Puducherry | 42.5 | 26 | 16.5 | 64 | 44.9 | 19.1 |
| Punjab | 51.7 | 42 | 9.7 | 71.1 | 56.6 | 14.5 |
| Rajasthan | 46.3 | 46.6 | -0.3 | 71.5 | 60.3 | 11.2 |
| Sikkim | 40.7 | 23.6 | 17.1 | 56.4 | 55.1 | 1.3 |
| Tamil Nadu | 48.3 | 44.4 | 3.9 | 57.4 | 50.7 | 6.7 |
| Telangana | 53.2 | 48.2 | 5 | 70 | 60.7 | 9.3 |
| Tripura | 61.5 | 54.4 | 7.1 | 64.3 | 48.3 | 16 |
| Uttar Pradesh | 45.9 | 51 | -5.1 | 66.4 | 63.2 | 3.2 |
| Uttarakhand | 46.4 | 46.5 | -0.1 | 58.8 | 59.8 | -1 |
| West Bengal | 62.3 | 53.6 | 8.7 | 69 | 54.2 | 14.8 |

**Supplementary Figures**

**Supplementary Figure S1**: Gender differences in IFA supplementation coverage among school- going adolescents across districts, India 2019-20


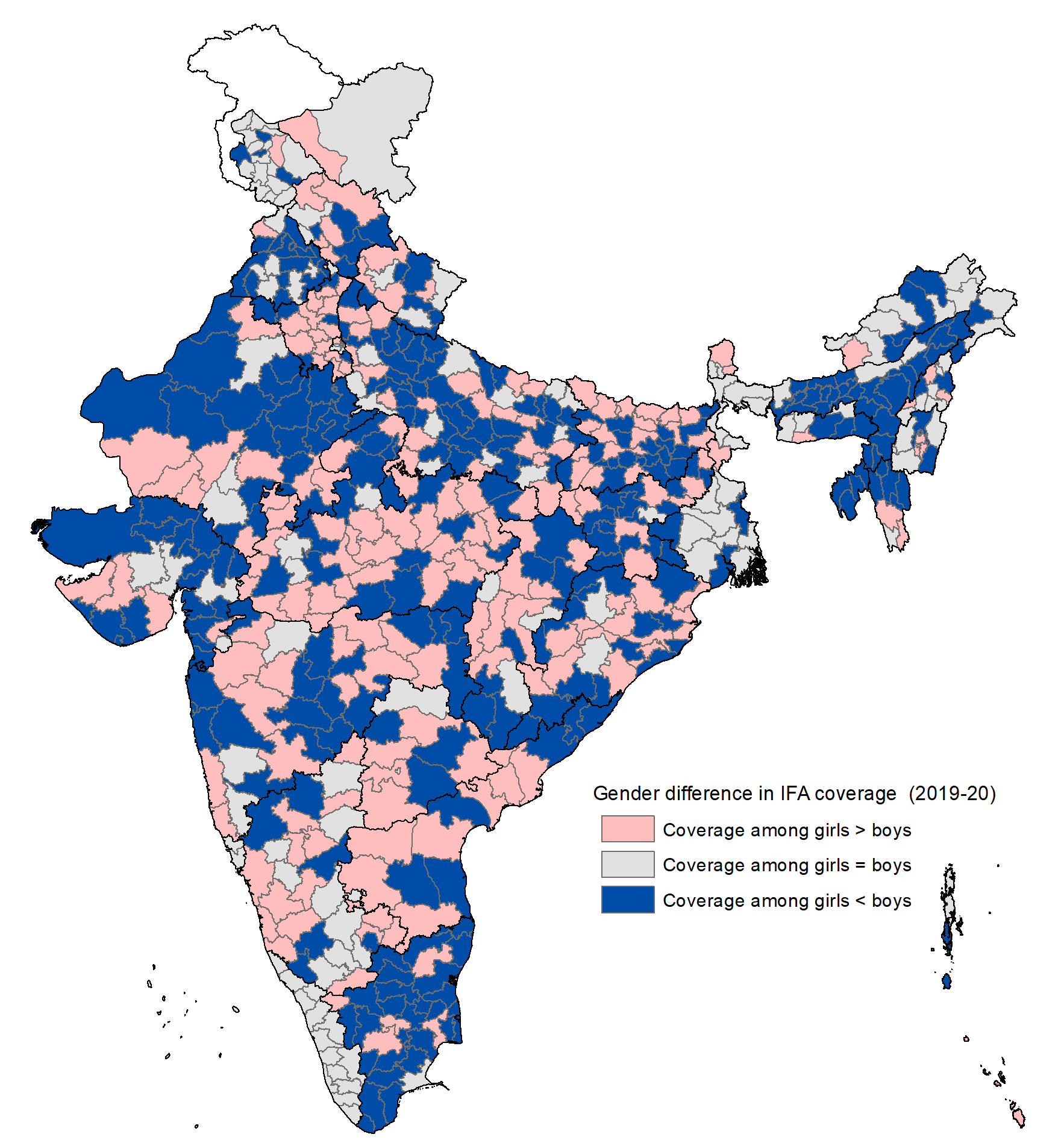


*Source*: Authors based on HMIS and AMB dashboard ([www.anemiamuktbharat.info](http://www.anemiamuktbharat.info))

**Supplementary Figure S2**: Direction of change in IFA 180 tablets coverage based on change observed between NFHS 2015-16 and 2019-20 and its comparison with HMIS 2017-18 and 2019-20

**Supplementary Figure S3**: Association of change in IFA 180 tablets coverage among pregnant women based on HMIS 2017-18 and 2019-20 and change in prevalence of anemia among pregnant women based on NFHS 2015-16 and NFHS 2019-21
